# TRIPI: A global dataset and codebase of the total resources in physical infrastructure encompassing road, rail, and parking

**DOI:** 10.1016/j.dib.2024.110387

**Published:** 2024-04-03

**Authors:** Martijn van Engelenburg, Sebastiaan Deetman, Tomer Fishman, Paul Behrens, Ester van der Voet

**Affiliations:** Institute of Environmental Sciences (CML), Leiden University, Einsteinweg 2, 2333 CC Leiden, the Netherlands

**Keywords:** Transportation, Construction materials, Urban mine, Spatial, Stocks, Network

## Abstract

Construction materials are associated with significant environmental and resource impacts. The circular use of materials already in use as stocks may provide an opportunity to reduce these impacts. We provide a dataset describing the potential global urban mine consisting of transportation infrastructure in an open database based on geospatial data from OpenStreetMaps. We reveal the significant opportunities of the embedded materials in this huge stock. With this Total Resources in Physical Infrastructure, or TRIPI, the database we provide easy access to a global dataset covering 175 countries and sub-regions, allowing researchers to select an area of study, and find the location as well as the material composition of the physical infrastructure. Material stocks are reported on a national level and commonly used regional aggregations. Material stocks are reported per kg, kg per capita, and kg per area; and for the physical type of infrastructure that is available in kilometres and area (km^2^). This dataset can be used in various research applications such as Material Flow Analysis, Material stock inventories, Country-level comparisons of infrastructure density, and others, and inform policy on harnessing the opportunities of the urban mine.

Specifications Table

**Table:** 

Subject	Renewable Energy, Sustainability and the Environment
Specific subject area	Establishing the composition of the urban mine of transportation infrastructure by measuring the global in-use stocks.
Type of data	Spreadsheet files containing country-level detail on infrastructure composition. Data presented in different stages, from raw data to filtered and processed.
Data collection	Data was acquired from OpenStreetMaps (OSM) by extraction through geofabrik.de. All data were downloaded and processed between February 2023 and March 2023. Data were collected on a country-level basis for 163 countries, for 13 countries where sub-national data were available this was taken instead. Pre-processing of the data was done by extracting the infrastructure data using the programs osmfilter and osmconvert within the Python-based software environment Spyder. The resulting files were used to calculate the dimensions and material content of the different transportation infrastructure types.
Data source location	Raw data used for this dataset is collected and stored centrally on https://planet.openstreetmap.org/ and has global coverage. The secondary processed data included in this dataset for transportation infrastructure has global coverage with local level spatial data quality.
Data accessibility	Repository name: A global dataset for the materials and in-use stock of transportation infrastructure, encompassing road, rail and parking. Data identification number: 10.5281/zenodo.10022371 Direct URL to data: https://zenodo.org/records/10022371 Github repository: https://github.com/MvEngelenburg/TRIPI Instructions for accessing the data: “for peer review only” – All data was included that resulted from the inventory of the OpenStreetMap data. The scripts should work straight away after downloading the necessary packages (the main one being PyrOSM), and after adjusting the directories in the Python files. We didn't include a raw OSM file on Github due to size limitation, but these can be downloaded from http://download.geofabrik.de/europe.html. It is easiest to test the code with a small file (i.e. Luxembourg, Benin, Greenland or similar). The downloaded .osm.pbf file needs to be in the same folder as the cmdlineprogram.py, osmconvert.exe and osmfilter.exe as these will be accessed to convert and adjust the file to the needed infrastructure elements (reducing file size).

## 1 Value of the Data

• Resource use has accelerated and is expected to remain high over the coming decades, resulting in broad environmental impacts. Construction materials in infrastructure have not been covered extensively in literature and the TRIPI dataset provides a comprehensive overview of the current size of the urban mine in infrastructure. As transportation infrastructure often has long lifetimes, it is crucial to have this baseline assessment available as an independent benchmark for decision-makers at all levels (e.g. municipalities, governments and global governing bodies) at a time when many investments are needed for the expansion and maintenance of the network. Knowing the volume and location of in-use stocks of resources will allow researchers to pinpoint hotspots of resource use. The presented data entails a prospecting of the urban mine in global transportation infrastructure and could be used in future research to assess the anticipated timing of the availability of these secondary raw materials.
• In spatial planning, urban planning, and circular economy research the efficient use of land and materials is being addressed in literature. A large part of the urban landscape is determined by the transportation infrastructure network. TRIPI allows researchers to determine the total land use of road networks, and explore optimal urban landscapes such as the ’15-minute city’. Transformations to efficient urban morphologies will lead to new material demand and outflows of the stocks. This dataset can be used to derive a localized inventory of the materials used in infrastructure and inform efficient reuse and recycling strategies.
• Researchers requiring a flexible geographic scope can use this dataset to include global, national or local elements of infrastructure in their analysis. Organizations that deal with global infrastructure such as the Global Infrastructure Hub, The World Bank and the United Nations can use TRIPI to add to their insights into infrastructure, materials and their implications for global welfare. Researchers within the field of industrial ecology can find detailed information on either the scope of the infrastructure (e.g. length, width, etc.) or the region-specific material composition of transportation infrastructure.
• Educators can use this dataset and related codebase to easily extract an area of interest for local GIS exercises and local insights. The current dataset gives flexible access to all global regions, so assignments can be made to compare different world regions on the scope of the transportation infrastructure. Detailed OSM data can sometimes be difficult to extract, as most available shapefiles only include a selected number of attributes. With the data, and codebase we provide easy access to experiment with the wide range of data available.
• This dataset can be the foundation for future scenario assessments related to resource use, as done for example by the International Resource Panel in their Global Resource Outlook [1,2]. In addition, it can improve coverage of material demand modelling in Integrated Assessment Models (IAMs) by providing the baseline of in-use transportation infrastructure. Combining this with scenarios on material use in other sectors such as buildings enables researchers to explore how circular economy strategies can be effective climate mitigation strategies, and vice versa. This can be of value for organizations such as UNEP, the World Bank, the European Union, national governments but even sub-national administrative units.

## 2 Background

Recent research on material use in buildings has made clear that construction represents a large share of total global material use. However, up until now the stocks of infrastructure have been underrepresented. Making it difficult to establish the total size of the urban mine. The developments in OpenStreetMaps and the global coverage of this type of open-access data present an opportunity to establish the quantities of materials in use in a spatially explicit way that is unprecedented. With this data inventory we aim to provide a highly detailed overview of transportation infrastructure not only covering the location but also specific materials used locally; to establish the current in-use stocks of materials. From the urban mining perspective, our data parallels the prospecting stage of traditional mining. This means mapping and quantification of the potential “reserves” which may become available for reuse and recycling in the future.

## 3 Data Description

**Fig. 1:**
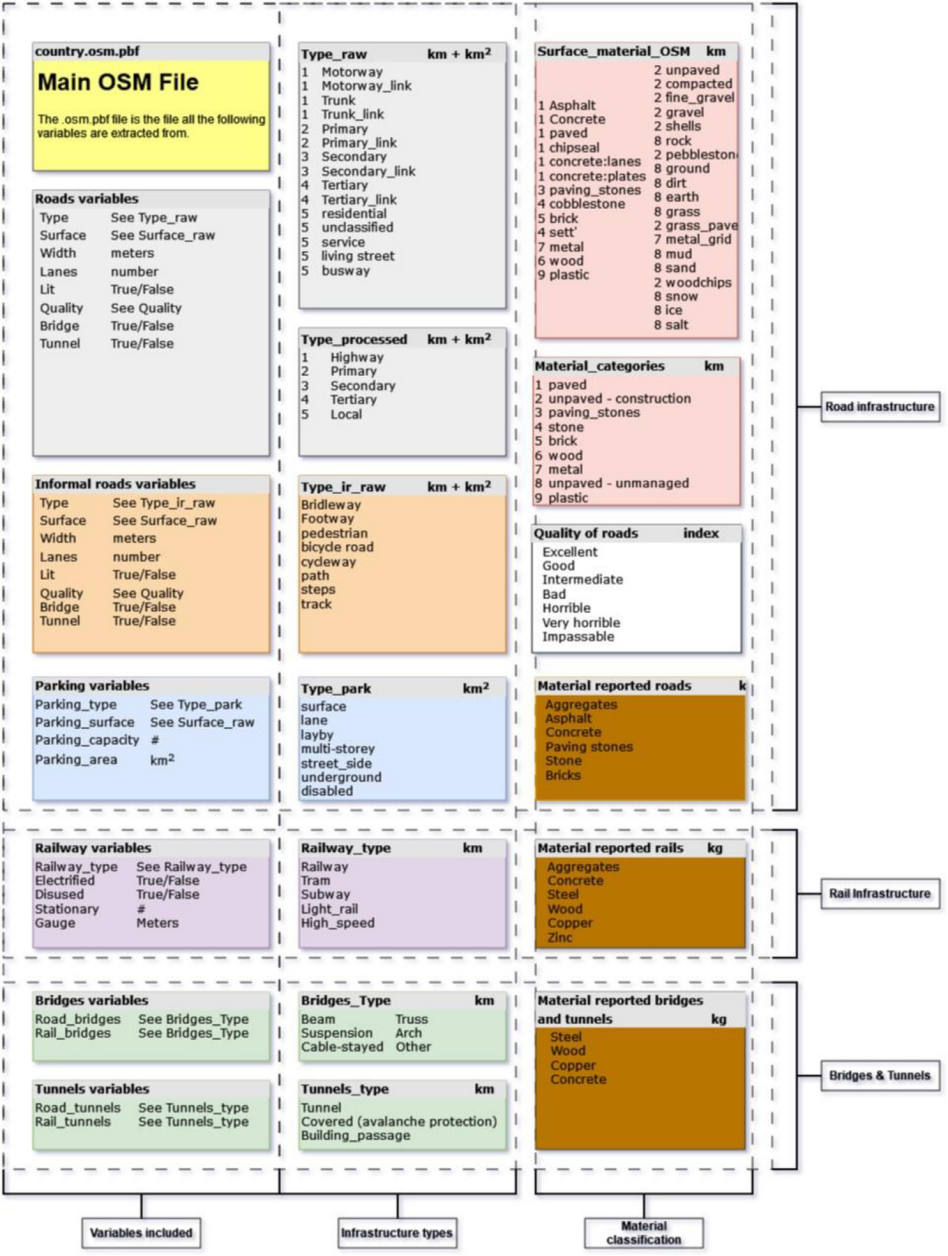
Overview of all the variables included in TRIPI. The left column includes the different aspects of each infrastructure elements. The second column shows the breakdown of the different types reported and the third column provides extra detail on the material classifications and the quality of elements where known. With the colours indicating the different infrastructure elements. Grey being roads, orange informal roads, purple railway and other rail based transportation infrastructure and green being bridges and tunnels. In red we depict the surface materials found as in OSM, and the final categories these materials are placed in for calculation of the materials. In white the quality of roads which is an index based on smoothness values found in OSM and in brown the final materials in kg we present in the TRIPI database.

The data contains information about the length of the transportation infrastructure, the total area in combination with width information, information on the material composition of the infrastructure elements, the quality of roads, on electrification of railways and the type of rail network. In addition, for bridges and tunnels, some information is available on the types that are most abundant. Fig. 1 gives an overview of all elements included in the TRIPI database. With the inclusion of the scripts on GitHub, data can be extracted for custom regions by making small changes to the codebase.

The data presented in the TRIPI database is based on the extraction of OSM data in February 2023. The data is divided into three different levels of regional definition National, the regions of the Global Roads Inventory Project (GRIP), and the regions of the Integrated Model to Assess the Global Environment (IMAGE), and is available on the GitHub repository. This regional definition is chosen as the country level is often used in analysis, GRIP regions as these represent seven global regions and is used for inclusion of the material intensity information from Rousseau et al. [3], and finally IMAGE as this is an IAMs where attempts are being made to cover materials within the CIRCOMOD project. Some sub-national regions are also included in the database, to show the potential of regional definition of the codebase.

**Fig. 2:**
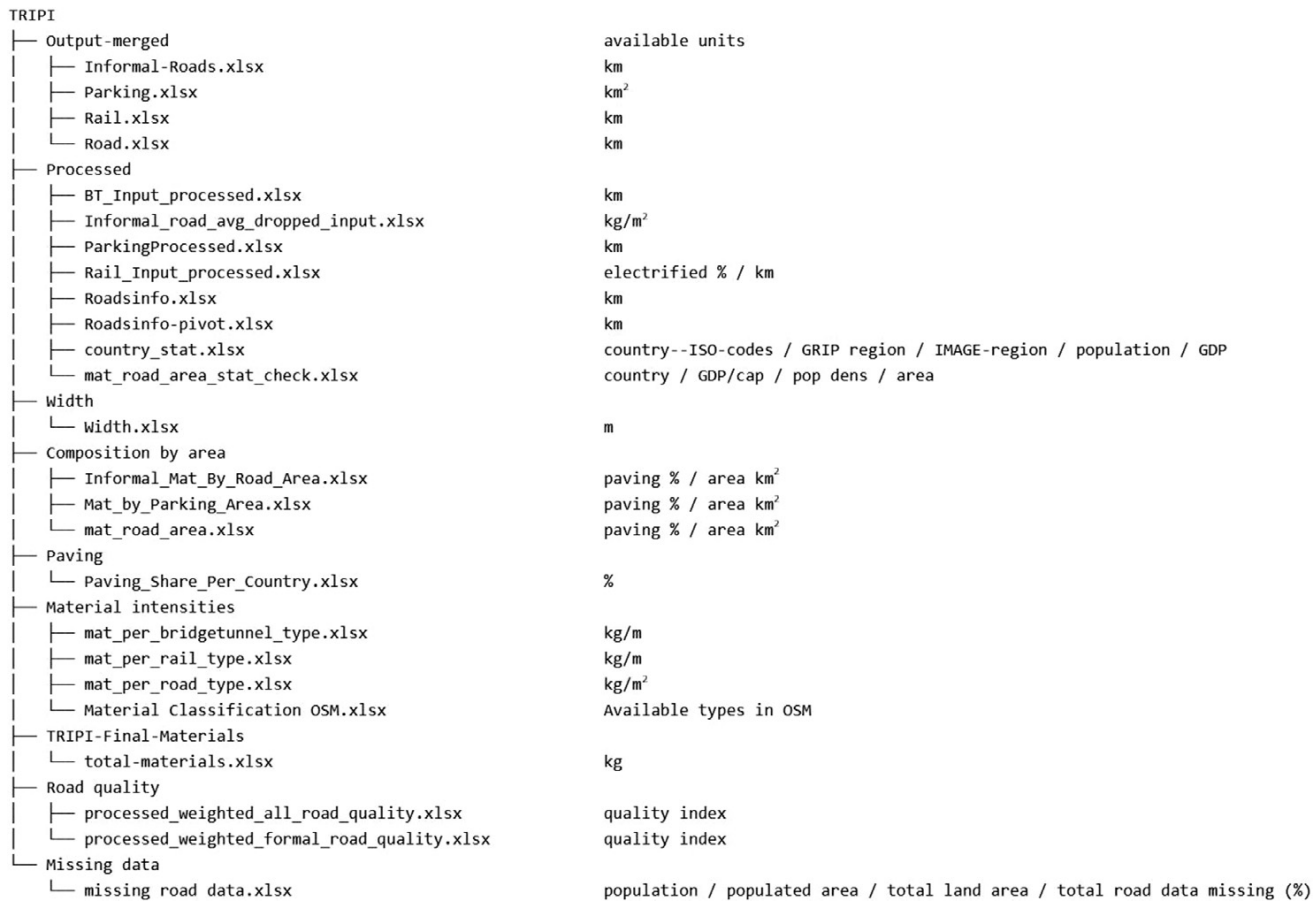
overview of the available folders in the TRIPI database and the units available for each file.

First is the raw data extracted from OpenStreetMap [4], available under the Open Data Commons Open Database License, directly per sub-region and per country. This data is available for 176 countries and is located in the folder “Output-merged”. For several countries the raw data is available on a sub-national basis, these are the USA, Canada, India, Indonesia, Japan, Russia, France, Germany, Great Britain, Italy, The Netherlands, Poland and Brazil. In Fig. 2 we show the types of infrastructure for which this raw data is available. Here the data is kept raw, excluding any form of data cleaning or completion. The second set of Excel files, located in the folder “Processed”, contains completed and cleaned data. In that folder, the files that have been processed using the processed.py script can be found.

**Fig. 3:**
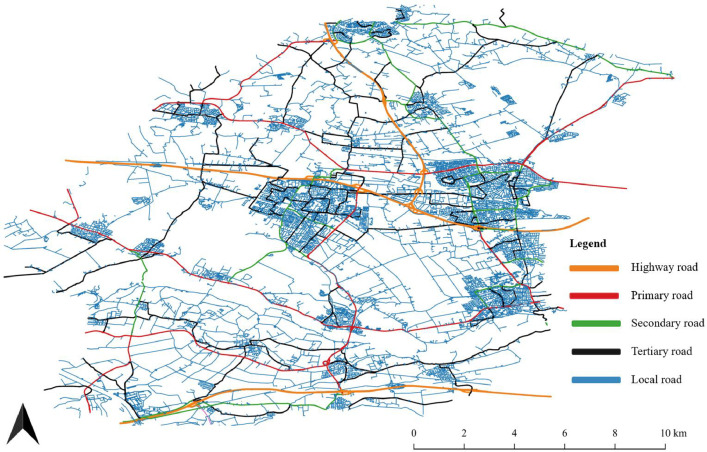
A snapshot of the Veenendaal region in the Netherlands showing the typology of the different roads and how each road is processed. Here the roads have been processed to the five types available in the material database. This map was made by extracting a smaller road network from the OSM data by overlaying it with a custom polygon for the Veenendaal region, Then this was loaded into QGIS where all the roads were labelled by colour.

The folder “Width” contains data on the width of roads per road type per country. In case of insufficient or incorrect data, a global average is taken. Within the folder “Composition by area”, the files including the processed material information reside. In Fig. 3 we show the road typologies as used for the final material calculation and are available within the database.

**Fig. 4:**
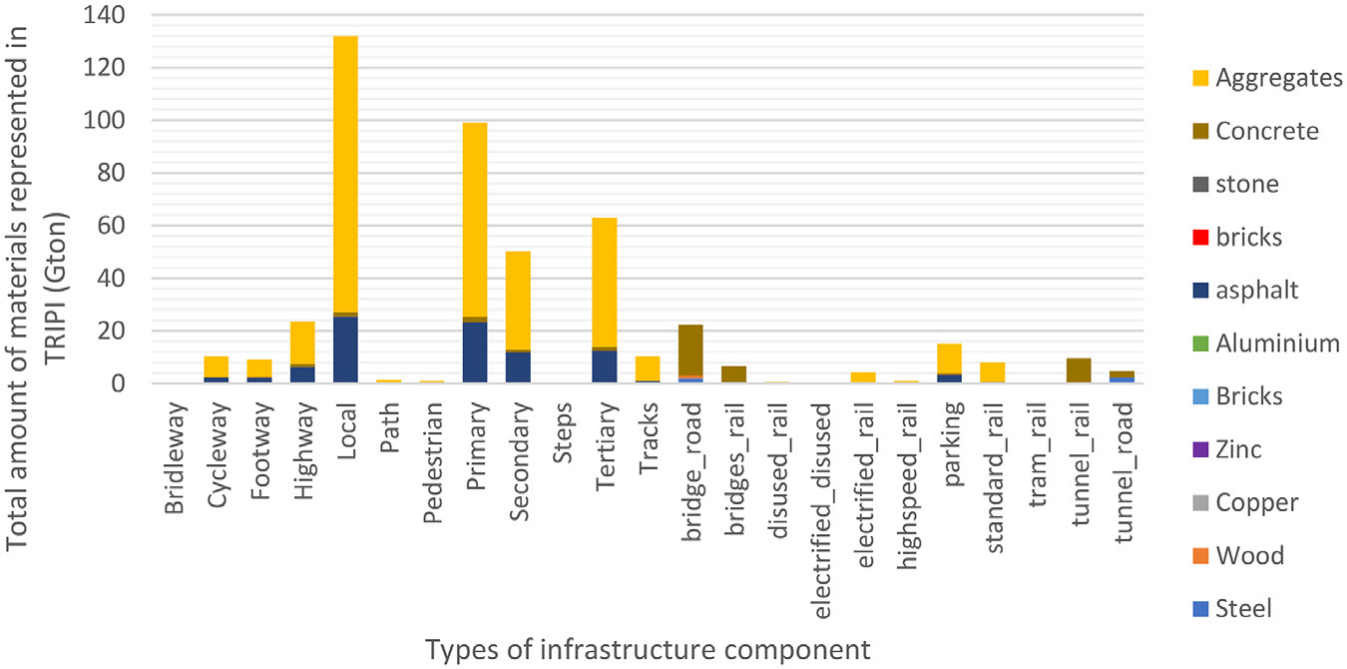
Breakdown of the total global material contribution per type of infrastructure available in the TRIPI database. The main bulk of materials is still in the road network with local, primary, secondary and tertiary roads being the bulk of the materials.

Within the paving data spreadsheet, we give an overview of the share of paved roads for each individual country based on the OSM data. The dataset presents the paving share, as well as the main material with which the road is paved, and what types of unpaved roads there are (i.e. dirt, grass, ice, or other road types). These paving data enable users to compare OSM-derived data with data from national statistics. In combination with the files from the folder “Material Intensities”, that contain data on particular materials per km of particular road types, it is possible to calculate the amount of materials embedded in the transportation infrastructure of any selected area. The result, the amount of materials embedded in transport infrastructure, can be found in the folder “Materials”. This folder contains the total amounts of materials in in-use stocks of infrastructure on different scale levels: country level, GRIP regions, and IMAGE regions). GRIP [5] and IMAGE [6] country placement categories are provided to facilitate easy incorporation into these models. Other regional aggregations are not provided in TRIPI, but can be easily made using the country-specific data. The Materials folder also provides an overview of the material per infrastructure type by providing the total materials for each type of infrastructure. For the breakdown of the different types see Fig. 4.

The folder “Road Quality” combines all the data available on road smoothness/quality. This is divided into two processed files for roads, and roads plus informal roads. Due to data limitations, the road quality data is only available for 164 countries. Finally, The folder “Missing Data” gives an overview of the populated areas that have no road information on OSM. The folder structure is also presented on GitHub including the scripts as described in the next section.

### 3.1 Material stocks description

In Fig. 4 we present a breakdown of the amounts of materials embedded globally in each type of transportation infrastructure from all represented countries in the database. It can be seen that the formal road network, being local, tertiary, secondary, primary and highway, is the largest share of materials. Please see the Excel file total-materials in folder TRIPI-final-materials. Aggregates are the largest material category, followed by concrete and asphalt. The different types of materials in different transportation infrastructure are available in the materials.xlsx file, per country.

**Fig. 5:**
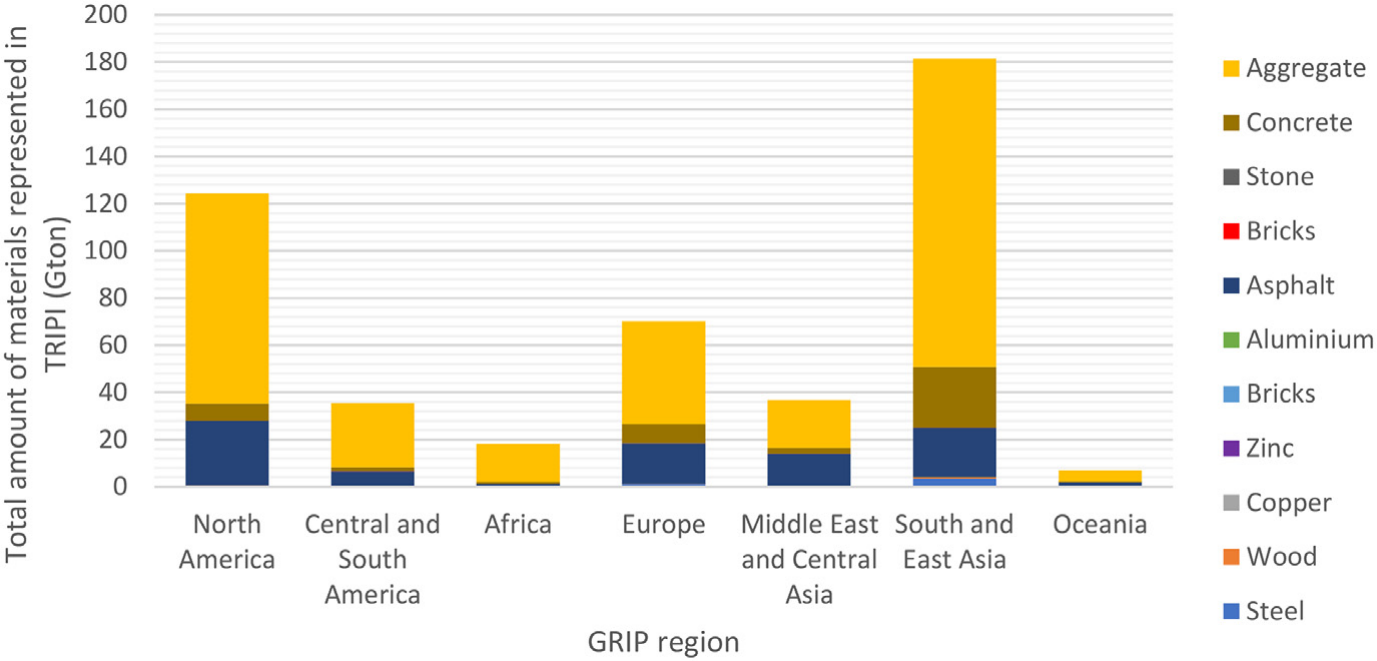
Breakdown of the total materials per GRIP region. These are the seven global regions available to compare different areas.

**Fig. 6:**
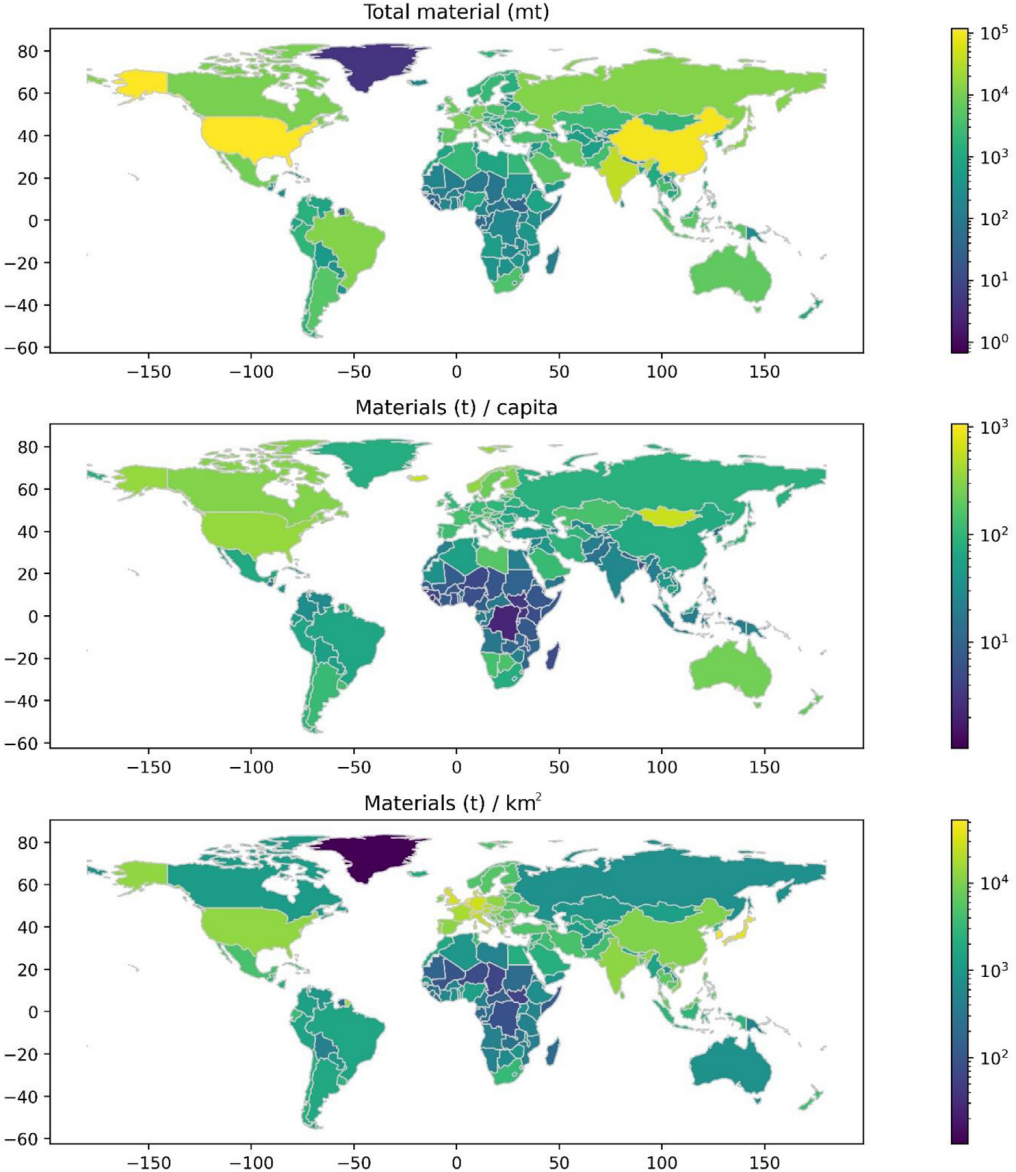
The coverage of the TRIPI database for all countries and regions showing the current in-use stocks transportation infrastructure for each represented country. We show this by total amount of materials (Mt). Materials per capita (t/cap) and materials per area (t/km^2^).

A different breakdown is presented in Fig. 5 by region, with the GRIP classification as an example. Fig. 6 shows the embedded materials by countries available in the TRIPI database. This level of detail is also available in the total_materials.xlsx for each country, Providing easy access to data for researchers to make country-by-country analyses.

## 4 Experimental Design, Materials and Methods

**Fig. 7:**
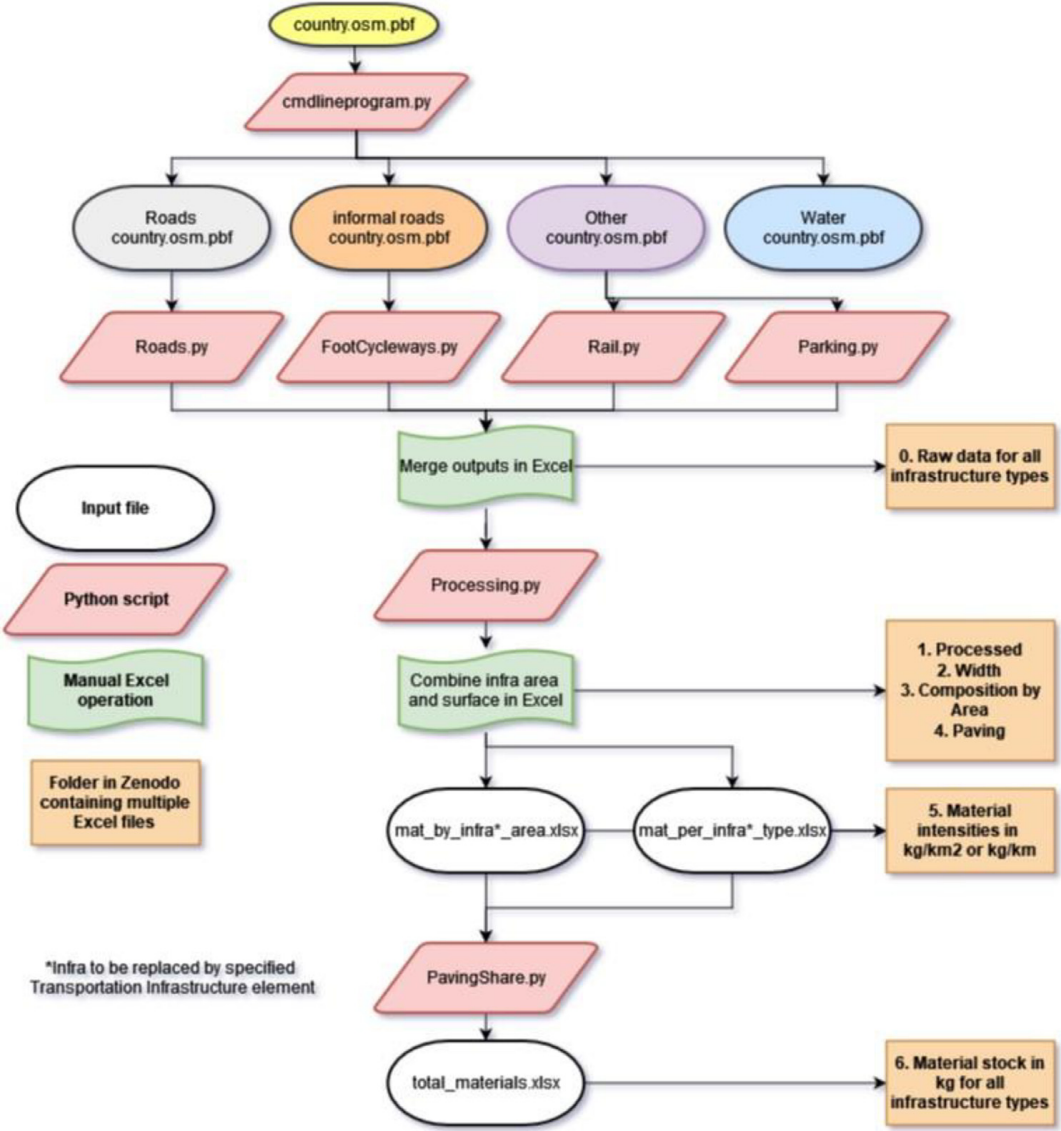
Overview of data extraction and processing steps.

Fig. 7 shows the procedural overview for extracting the data made available in the TRIPI dataset (and available on Zenodo). Additionally, the .osm.pbf files are available on Zenodo due to Github data storage limitations. For planned updates of the database, please see the section “TRIPI roadmap” below. We used PyrOSM [7], a Python module which extracts, sorts and allows for manipulation of .osm.pbf data downloaded from GeoFabrik [8]. The data from Geofabrik is a subdivision of the planetary OpenStreetMap data at country and sub-country levels, accessing the whole OSM planet file is possible but due to the large file size is not as computationally efficient to work with. Pre-processing of these files is necessary as the required computing power increases with file size. This pre-processing is done by splitting the GeoFabrik files up into the attributes that are of interest (roads, railways, etc.), leaving smaller files for each infrastructural element. This was done using the applications osmfilter and osmconvert within the script cmdlineprogram.py available on the TRIPI Github. With this script each country-level.osm.pbf file is loaded and split into four categories: formal roads, informal roads (containing walkways, bike paths and tracks), other and waterways. In the current dataset the category ‘other’ encompasses all elements not included in formal roads, informal roads and waterways and thus includes railways and parking. After these pre-processing steps, we extracted each separate infrastructure element for each country and calculated all aspects of interest.

We differentiate formal and informal roads because informal roads are often not included in national road statistics. This allows for easier comparison with data from national statistics. Formal roads consist of motorways, primary roads, secondary roads, tertiary roads, and local roads (residential, unclassified, service, and living streets). Informal roads consist of bridleways, footpaths, cycle paths, and tracks. This classification is then also used in the material stocks calculations. First, the total length of all roads is calculated per road type, then we multiply this by the width per road type to calculate the total surface of the road network. For countries where width data is missing, incomplete or incorrect, we use a global average. For the calculation of the rail network, we assume each line element in OSM to be a single track (we visually inspected multiple data points to verify this). We include standard rail, electrified rail, tram, subway, light rail, metro, abandoned, construction, planned, disused, and razed (rail infrastructure where it is still visible that it used to be a railway track, but the materials have been removed). The razed infrastructure category is included for identifying where these locations are, but has no impact on the total materials. For bridges and tunnels, we calculate the length, and we extract the bridge type from the column ‘bridge:structure’ using the roads.py script. The results are found in Output-merged – Roads.xlsx. For the different bridge types, we extract the most common bridge types used globally (beam, cable-stayed, arch, etc.). For tunnels, we included all tunnels that are labelled as ‘Tunnel=yes’ and excluded the label ‘Tunnel=building_passage’ as these are not part of the official transportation infrastructure network. We also collect the data that is included on the type of parking infrastructure. Including the types surface, parking, multi-storey garage, underground, street-side, carports and others. Furthermore, we extracted the data on capacity per parking area to find the total parking capacity per country. This data is all extracted using the Parking.py script, after which all spreadsheet files are exported containing all elements per country (and for larger regions: per sub-region). A disadvantage is that we lose the high resolution of the spatial data. Nevertheless, the TRIPI database provides access to the data, making it widely available for parties not necessarily interested or able to perform a high-resolution analysis. In addition, this level of detail is lost anyway when correcting for missing data on OSM which we address in the next section.

### 4.1 Completeness of network

As the data from OpenStreetMap alone does not provide full coverage for all infrastructural elements [9], some calculations are made to ensure the dataset represents the full length of the transportation infrastructure. Previous studies [9] have compared the completeness and total road network to national statistics and found that OSM has reasonable coverage but still misses data. We used a dataset that checks for missing elements in OSM called disaster.ninja [10]. Kontur gathers data in an attempt to map out potential risks in case of a disaster. This is done by mapping out populated areas and overlaying them with OSM data for roads and buildings. They find that roads and buildings data are missing if there are people living in that area but none of these features exist in OSM. This is reported per administrative boundary ranging from country level to municipal level. We use this information on a national level to estimate how much of the road network is missing per country. This is reported in area and %, and can be found in the folder ‘Missing data’. By integrating this within the TRIPI dataset we provide information both on the observed data and on the estimated complete road network.

For parking, data completion is hard to establish and data is lacking compared to road and rail. In TRIPI we provide the parking data based on what is available on OSM. For a check on completion, we combine this data with the information found on missing road data from disaster ninja and look at countries that have almost no missing road data (<1%). Then we calculate the ratio of the parking area compared to the total road network area. This ratio (6.4% of the area of the total road network) is then applied to other countries to provide a first estimate for the amount of materials in parking infrastructure. For rail infrastructure, the only comparison that currently is possible to assess the completeness of OSM data is a comparison with national statistics. Comparing the data with the International Union of Railways (UIC) which provides an overview of the length of the rail network per country [11] we observe a larger rail network in OSM than is reported by UIC by a factor of 1.99. The difference is likely attributed to the definition of the rail line, where we calculated each individual line for the sake of material calculation, and the IUC calculates the operational rail length irrespective of the number of parallel tracks. Here we assume the network found on OSM to be complete.

### 4.2 Material stock calculations

Material stock estimates for the different types of transport infrastructure are calculated in the database in two ways. First, we collected data on the material composition of different elements of transportation infrastructure. We reviewed available literature on material intensities [3,[12], [13], [14], [15], [16], [17], [18], [19], [20], [21], [22], [23]]. For rail infrastructure, bridges, and tunnels, intensities from the literature are used which can be found in the folder material intensities. The material intensities for rail infrastructure are all expressed in kg/km and applied based on the type of infrastructure observed. For example, for standard rail the intensity of rail kg/km is taken, but for the share of electrified rail we apply the material intensity of electrified rail. For tunnels and bridges, the sources that are used include the kg/km intensities for the supporting structures. Second, for roads and parking areas, OSM provides information on the road or parking surface. This is used to establish the type of paving material in the infrastructure. This is where this dataset provides a unique perspective by not only accounting for paved roads but the actual material that the road is paved with following local observations. This is combined with an extensive dataset from Rousseau et al. [3] which provides material intensities for the paved road network for different global regions and different road types, we can calculate the total materials used in the global transportation infrastructure. As the surface information is not in all cases commonly found in literature, additional data was found on materials specifically for bricks, stone and paving stones which will be addressed in the next paragraphs.

In this database, we rely on literature sources for the material intensity of flexible and rigid paving. Flexible paving – more commonly known as asphalt – consists of two main components, bitumen and aggregates. Rigid paving is most frequently used for concrete paving, metal and wood can also be found as a rigid paving surface but these are most common in bridges. In this dataset, rigid and flexible paving are labelled as paved roads which is a type used in OSM. Then the extensive road paving study from Rousseau et al. [3] is used for quantifying the materials in these paved roads. In that study, the material intensity used is dependent on the share of flexible and rigid pavement found in national data sources and literature and applied to each GRIP region. This information is then applied to the TRIPI database per GRIP region for roads tagged as paved.

### 4.3 Brick roads

Bricks are available in various sizes, and standard dimensions differ per country and within a country. We have chosen to use an average of 10 countries which are the common variations for those countries, but within each country and for other countries other variations are available. The reason that we decided to take an average of these 10 countries is that these cover many different regions while also showing there are similarities in the brick dimensions. For example, in the Netherlands twelve different dimensions are available for road bricks [24], but no data is available on what type is most common. Unfortunately, most literature on bricks is outdated. So we have to rely on standardized brick dimensions. We chose to use an average of the ten countries available as these have good global coverage. As more data becomes available it will be incorporated into our future revisions of the TRIPI database. The final dimensions are the average of the values found in Table 1.

**Table 1:** Overview of commonly found dimensions of bricks. Adapted from Brick Sizes, Variations and Standardisation | Scotland's Brick and Tile Manufacturing Industry (2023).

Standard	Metric (mm)
Australia	230 × 110 × 76
Denmark	228 × 108 × 54
Germany	240 × 115 × 71
India	228 × 107 × 69
Romania	240 × 115 × 63
Russia	250 × 120 × 65
South Africa	222 × 106 × 73
Sweden	250 × 120 × 62
United Kingdom	215 × 102.5 × 65
United States	194 × 92 × 57

Taking the values from Table 1 results in the final dimensions of 229.7 mm rounded up to 230 for the length. 109.55 mm rounded to 110 mm for the width and 65.5 mm rounded up to 66 mm for the height. The brick dimensions we use in TRIPI are 230×110×66 mm. Using these dimensions to get an area of 1 m^2^ we find 39.52 bricks per m^2^ which we round up to 40 bricks. The weight of bricks estimates also range between 1400 – 2400 kg/m^3^ [25], [26], [27]. Here we use the average of the most common brick types which are: Flettons, London stock, Red facing, sand cement, and sand-lime [26]. Resulting in 1.867 tons/m^3^ for bricks. With 40 bricks in 1 m^2^ and a height of 66 mm this results in 124.7 kg/m^2^ for brick roads.

### 4.4 Sett, cobblestone or stone paving

For sett paving, there is less standardization of the dimensions, and although commonly used in cube forms there are wide variations available. We use a commonly found dimension of 100×100×100 mm, resulting in 100 setts or cobblestones per m^2^. For densities, the variety of materials used for this type of paving is wider than for bricks. Basically, all types of stone can be used for setts or cobbles, but granite, sandstone, and limestone are the most commonly available. Limestone has a density of 2700 kg/m^3^, sandstone has a density of 2100 - 2400 kg/m^3^, and granite 2600 – 2800 kg/m^3^ [25,27,28]. Because the distribution of use of these different types is not known we assume a weight of 2550 kg/m^3^ by averaging the values for this type of paving. With a height of 100 mm and 100 stones per m^2^ this results in 255 kg/m^2^ for sett, cobblestones, or other stone paving. We compared our results to Nguyen et al. [29] and Garilli & Giuliani [30]. Nguyen et al. [29] mention 216.45 – 331.20 kg/m2 but this includes gravel, while Garilli & Giuliani [30] mention the different heights of stone paving which range from 100 to 150 mm. With this comparison, and the scarce literature on stone paving we assume the value of 255 kg/m2 until better data becomes available.

### 4.5 Paving stone

Paving stone broadly consists of three types of materials in road construction, concrete paving stone, natural stone, and brick paving stone. Brick and natural stone have been mentioned before, and we assume the same values for these. The density of concrete is between 2240 and 2400 kg/m^3^ [28]. The dimensions used are commonly similar to the dimensions used for brick roads due to standardizations of road construction practices. We assume the same dimensions as bricks, but with the density of concrete, it would be between 149.61 kg/m^3^ – 160.30 kg/m^3^. This averages to 154.96 kg/m^3^ which we round to 155 kg/m^3^ for concrete paving stone.

The remaining factor is the distribution of the types of these materials used globally. Currently, there are no reliable global estimates available on the share of materials used in paving stones except for a handful of market estimates stating around 60% concrete, 30% natural stone, and 10% bricks [31,32]. No studies were found to confirm these numbers, but some data was found in OSM that provides some information. Of the 3,049,935 data points for paving_stones in the OSM data, 2913 mention the material that the paving_stones consist of [4]. As data on the material composition of paving stones is limited, we apply the shares found in OSM to all paving stones (Table 2).

**Table 2:** Material information found in OSM on paving stones.

Material	Count	Share
Concrete	2014	69,13%
Natural stone	788	27,05%
Brick	111	3,81%
Total	2913	100%

For road paving, we multiply the area (km^2^) with the material intensities applicable to each road or parking type. For rail infrastructure we multiply the length (km) with the material intensities found, this is also true for all bridges and tunnels. By doing this we provide a global material intensity average for rail, bridge and tunnel infrastructure elements.

### 4.6 Quality of roads

One further inclusion in the dataset is the quality of the country-level road network. The OSM coverage on road quality is still far from complete, but we present a first estimate of the road quality of a country by using the data that is available. We have done this by taking the indicator ‘smoothness’, and assigning values ranging from 1 to 7 to the most common labels that are used for this description. The labels included are shown in Table 3.

**Table 3:** Description of the most common road quality indicators as found in OSM, with the value that is assigned to each category. The categories are bad to excellent and can be used for describing the quality/state of paved roads. The indicators impassable to intermediate can be used for unpaved roads where intermediate is a high-quality unpaved road.

Smoothness indicator as found in data	Description as adopted from OSM	Assigned value
Impassable	Roads that used to be passable, and can still be recognised as a road but impassable for wheeled vehicles	1
Very horrible	Very badly damaged roads, only passable for specialised vehicles such as tractors, ATV or tanks.	2
Horrible	Only unpaved roads with damage, potholes and ruts.	3
Bad	Heavily damaged paved road, in need of maintenance. Average speed is less than 50% of what it would be on a smooth road. Good unpaved roads are included here	4
Intermediate	Road showing damage or previously repaired damaged. Travelling at full speed is not always recommended. Best possible classification for an unpaved road.	5
Good	in good condition, but paving showing cracks and/or wear	6
Excellent	Road in new condition with smooth paving and seamless connections Road	7

In the TRIPI dataset, we present all roads that contain data on this indicator. For the country level, we aggregate all information available to a weighted indicator and present it for different regional scales. This is done by calculating the total length for each value found per type of road in kilometre. Each smoothness indicator is multiplied by the length and assigned a value of 1–7 after which we divide by the total kilometres. This results in a value between 1 and 7 and represents the quality of the type of road, and when done for all roads, in an average quality index for the whole network within a country.

Due to data limitations most sub-country regions do not have enough data points. In addition this data is lacking for parking infrastructure, and thus not yet provided in TRIPI. For railways this data is currently not available, so it is not part of the current dataset. When and if such data becomes available it will be added to the TRIPI database.

### 4.7 Comparison with other data sources

We compared TRIPI with different global inventories. Table 4 shows that TRIPI covers more roads than others, Partly due to the coverage of informal roads. We cover all materials used in infrastructure, not only the official road networks. We see alignment with the study by Wiedenhofer et al. [23], although the typologies differ and we include more km and materials because of the accounting for missing data. A large share of this is in aggregates as the missing data is usually in underrepresented regions with mostly unpaved roads. In addition, the difference can be caused by the applied material intensities applied for the different infrastructural elements, and the different calculation of total road area. For Rousseau et al. [3] this material estimate includes paved roads and not unpaved roads, bridges or tunnels.

**Table 4:** Comparison to other extensive infrastructure data.

Source	Total road coverage (km)	Road material stocks (Gton)	Road types included	Reported years	Regional coverage
TRIPI dataset	82,452,154	440.29	Roads, bridges, tunnels, parking	2023	Flexible
Wiedenhofer et al. [23]	71,843,828	295.31	Roads, bridges, tunnels	2022	National
CIA Factbook [33]	39,297,688	n.a.	n.a.	Various	National
Meijer et al. [5]	21,600,000	n.a.	n.a.	Various <2015	National
Rousseau et al. [3]	23,570,000	182.61 – 331.87	Paved roads	Various	National

### 4.8 TRIPI roadmap

OSM data is continuously updated, and some aspects are currently not yet included such as power, water and communications infrastructure, which could be added to TRIPI in the future. The aim is to update TRIPI at annual intervals. Primarily these will be updates where the increasing coverage of OSM is included, but coverage can also be extended to the mentioned other types of infrastructure. For the coming update, we aim to streamline the extraction of data by minimizing any need for manual calculation steps. In addition, we are exploring the option of providing more sub-national data. Future TRIPI updates may require other data inputs besides OSM, as data completeness is likely a limiting factor for infrastructure types beyond the ones currently covered.

## Limitations

As described in the previous section, not all data for each country is complete and the calculations for missing infrastructure are based on national-level estimates. Regionally there may be differences in completeness, and this should be taken into consideration when applying TRIPI data. A further issue is that the voluntary input the OSM data depends on might have been labelled incorrectly. Furthermore, the material intensities we use in the TRIPI database are a representation based on relatively few literature sources, and local compositions might differ widely from what is presented. This is a well-acknowledged challenge [34], [35], [36], [37]. Nevertheless, it provides a baseline for further analysis and comparison. There are currently no large-scale checks to verify the OSM data. The comparison we have included to compare the total materials to other studies such as Wiedenhofer et al. [23] or Rousseau et al. [3] is a form of validation, but these studies also at least partially involve OSM as input. From the method we used by visual confirmation using satellite imagery and accounting for road completeness, we address this issue as much as possible.

## Ethics Statement

The authors of this publication follow the ethical requirements and confirm that the current work does not involve human subjects, animal experiments, or any data collected from social media platforms.

## CRediT authorship contribution statement

**Martijn van Engelenburg:** Conceptualization, Methodology, Writing – review & editing, Data curation, Writing – original draft. **Sebastiaan Deetman:** Conceptualization, Writing – review & editing, Supervision, Funding acquisition. **Tomer Fishman:** Writing – review & editing, Supervision, Funding acquisition. **Paul Behrens:** Writing – review & editing, Funding acquisition. **Ester van der Voet:** Conceptualization, Writing – review & editing, Funding acquisition.

## Data Availability

TRIPI: A global dataset and codebase of the Total Resources In Physical Infrastructure encompassing road, rail, and parking. (Original data) (Zenodo).
